# Immunohistochemical Analysis of Oral Spindle Cell Hemangioma 

**DOI:** 10.30476/dentjods.2024.101499.2305

**Published:** 2025-06-01

**Authors:** Amit Mani, Manas Bajpai, Saurabh L Sabnis

**Affiliations:** 1 Dept. of Periodontology, Pravara Institute of Medical Sciences, Rural Dental College Loni (Maharashtra) India.; 2 Dept. of Oral Pathology and Microbiology, Pravara Institute of Medical Sciences, Rural Dental College Loni (Maharashtra) India.

**Keywords:** Hemangioma, Immunohistochemistry, Hemangioendothelioma, Palate

## Abstract

Spindle cell hemangioma (SCH), formerly called “spindle cell hemangioendothelioma”, is a rare benign histological variant of hemangioma characterized by the presence of two contrast zones, the first zone exhibits large dilated cavernous space with slit-like vascular spaces may show clear endothelial vacuoles resembling fat cells. SCH is often considered as pseudosarcomatous entity; it imposes a diagnostic challenge for oral pathologists due to its resemblance with Kaposi sarcoma. A total of 13 cases of SCH have been reported in the head and neck region to date and only 6 cases have been reported inside the oral cavity. We present a rare case of SCH located on the hard palate, which imitated Kaposi's sarcoma on histopathological examination. The expressions of various markers including EGR, CD 31, and HHV 8 yielded the final diagnosis of SCH. The markers EGR and HHV 8 have never been used in intraoral SCH before to the best of our knowledge; hence, the present report highlights the use of immunohistochemistry for the diagnosis of SCH.

## Introduction

Spindle cell hemangioma (SCH) is a benign vascular neoplasm that chiefly affects the skin, its occurrence in the soft tissue of the head and neck is rare [ 1 ]. An exhaustive review of the literature revealed that only 13 cases of SCH have been reported in the head and neck region to date [ 2 ]. Intra- orally, its occurrence is exceedingly rare with only 6 cases reported in the literature [ 2 - 3 ]. Here we report an additional case and probably the second case of SCH located on the hard palate, which was preliminarily diagnosed as benign minor salivary gland tumor and malignant tumor of vascular origin on histopathological examination. The final diagnosis of SCH was rendered with the correlation of histopathology and immunohistochemistry. An exhaustive literature review revealed that immunohistochemical markers including CD 31, CD 34, PCNA, factor VIII, vimentin, and HAM 56 have been used for the diagnosis of SCH; however, the markers HHV 8, WT 1, and EGR have never been used before. 

## Case Presentation

**Figure 1:**
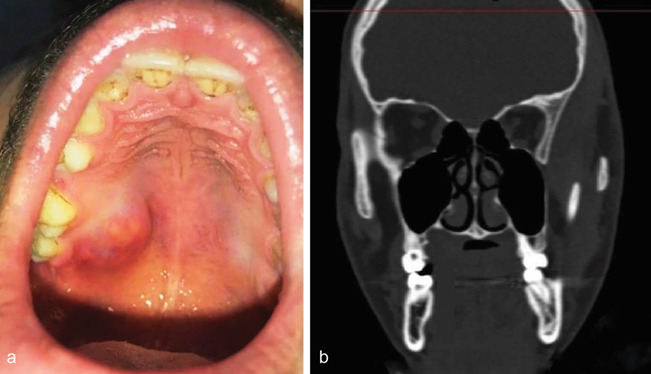
**a:** Clinical picture of the lesion, **b:** CT scan of the patient

A 28-year-old male presented to our institution for the evaluation of a localized, painless growth on the right back region of his palate for 2 years. The personal history, family history, and past medical history of the patient were non-contributory to the presenting symptom. The extraoral examination did not show any facial asymmetry. Intraoral examination revealed a dome-shaped growth of the posterior palate measuring about 3×2(cm) in dimension on the palatal surface of teeth number 16 and 17. The color of the swelling was slightly red in comparison to the adjacent mucosa (Figure 1a). No discharge or sinus was noted. On palpation, the swelling was found to be soft and non-tender. The provisional diagnosis of benign salivary gland tumor was given. Computed tomography revealed that the swelling was not associated with the underlying bone (Figure 1b).

**Figure 2:**
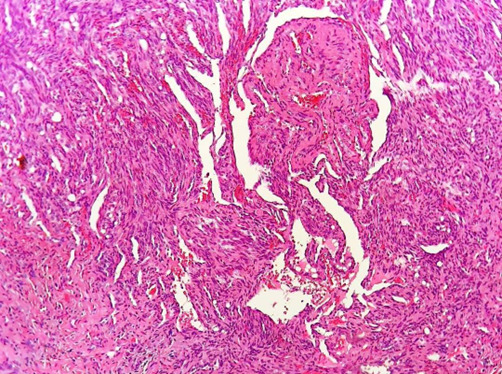
Dense sheet of spindle shaped cell along with a thrombi inside large vascular spaces (Hematoxylin and Eosin stain 20×)

**Figure 3:**
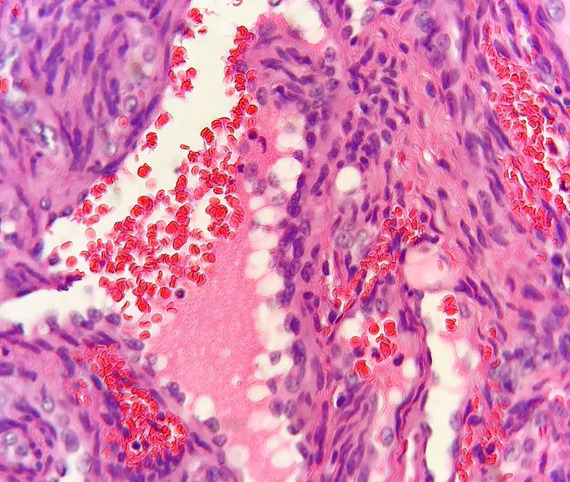
Numerous extra– vasated RBCs (Hematoxylin and Eosin stain 40×)

**Figure 4:**
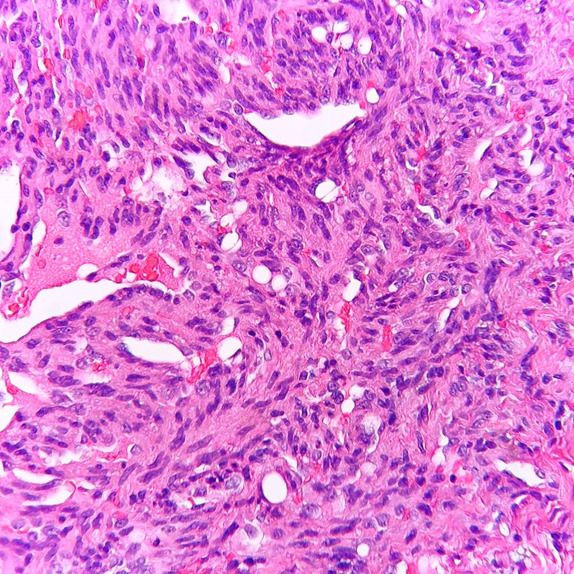
Biphasic patterns of cells composed of numerous clear endothelial vacuoles resembling adipocytes with sheet of spindle shaped cells with short fascicles (Hematoxylin and Eosin stain 40×)

**Figure 5:**
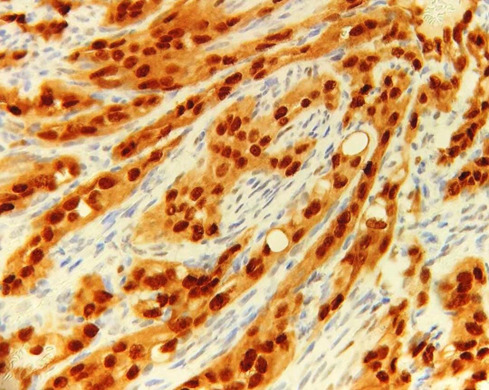
Positive expression of EGR for tumor cells

**Figure 6:**
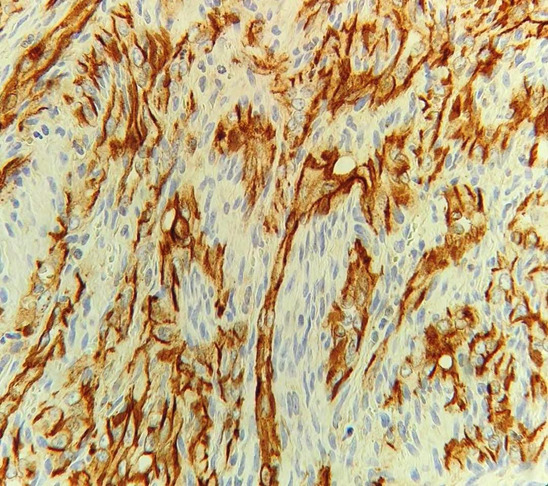
Positive expression of CD 31 for endothelial cells and negative expression for spindle shaped cells

**Figure 7:**
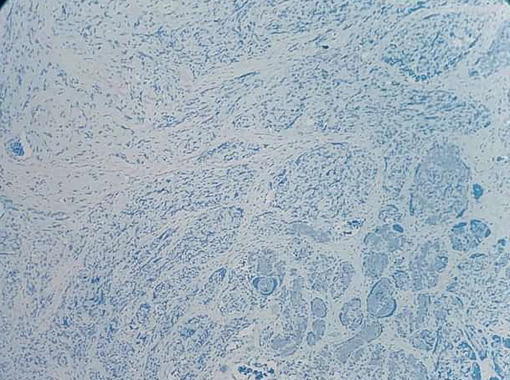
Negative expression of HHV 8 for tumor cells

An incisional biopsy was performed under local anesthesia including greater palatine nerve block with local infiltration. The excised tissue was sent for histopathological evaluation. The hematoxylin and eosin-stained soft tissue section revealed a dense sheet of spindle-shaped cells arranged in short fascicles along with cavernous space filled with thrombi (Figure 2) with the collection of numerous extra–vasated RBCs (Figure 3) along with numerous clear endothelial vacuoles resembling adipocytes (Figure 4). Mitotic figures were also noted in some places. Based on histopathological features, a diagnosis of malignant vascular tumor preferably Kaposi's sarcoma was given, to confirm the diagnosis immunohistochemistry including the markers EGR, CD 31, and HHV 8 was performed. The immunohistochemical expression of ERG and CD 31 were positive for endothelial cells (Figures 5-6) confirming the vascular nature of the tumor, the expression of HHV 8 was negative for tumor cells (Figures 7) excluding the possibility of Kaposi’s sarcoma and Wilm’s Tumor. After the correlation of histopathological features and expression of various immunohistochemical markers, the final diagnosis of SCH was given. The lesion was completely excised under general anesthesia. The follow-up period of one year was uneventful.

## Discussion

SCH was first described by Weiss and Enzinger [ 4 ] as “spindle cell hemangioendothelioma”; a vascular tumor characterized by areas resembling capillary hemangioma and Kaposi's sarcoma.

The tumor was initially considered as an intermediate grade tumor; having the biological behavior between hemangioma and angiosarcoma [ 4 - 5 ]. The World Health Organization (WHO) renamed it SCH in 1986 depicting its benign course [ 6 ]. Spindle cell hemangiomatosis is the term that describes multiple SCH, this term was first coined by Perkin and Weis [ 7 ] since then, the term SCH has been used exclusively for solitary lesions.

SCH is rare in the soft tissue of the head and neck with only 13 cases reported to date. Inside the oral cavity, it is exceedingly rare with only 6 cases found in the literature (Table 1). The etiology of SCH is not clearly understood; few studies have shown its association with Maffucci’s syndrome and Ollier’s syndrome [ 5 , 7 ]. The occurrence of SCH has been specifically noted with the mutations of IDH1 or IDH2 . The resemblance of SCH and angiomatosis was described by Perkins and Weiss [ 7 ] due to the presence of abnormally engorged vessels, herniation, and intraluminal webs. They suggested that SCH is a benign vascular tumor and the biphasic nature of the tumor is due to the alteration in blood flow.

**Table 1:** Review of previously reported cases of SCH inside oral cavity (NA- Not available)

S.NO	Author	Age/ gender	Location	Provisional diagnosis	Immunohistochemistry
1	Tosios *et al*. (1995) [4]	12/F	Mandibular vestibule	Hemangioma	The tumor cells show positive expression for HAM 56 and Vimentin, variable reactivity for factor VIII and negative expression for PCNA
2	Ide *et al*. (2004) [5]	55/M	Palate	Pyogenic granuloma	NA
3	Lade *et al*. (2005) [6]	25/M	Posterior pharyngeal wall	Synovial sarcoma	Not performed
4	Sheehan *et al*. (2007) [7]	44/M	Buccal mucosa	Vascular tumor	Positive expression of tumor cells for CD 31 and CD 34
5	Chavva *et al*. 2015) [9]	33/M	Floor of the mouth	Minor salivary gland tumor	Positive expression of tumor cells for CD 31 and CD 34 markers
6	French *et al*. (2016) [10]	52/F	Tongue	Not mentioned	NA

Intra- orally SCH tends to mimic various other clinical entities including pyogenic granuloma, hemangioma, peripheral giant cell granuloma, salivary gland tumors, and so on [ 3 ]. In the present case, the provisional diagnosis was benign salivary gland tumor as the lesion was associated with the palate. Histologically, SCH shows a lobular pattern of blood vessels with biphasic architecture including vascular spaces and solid connective tissue stroma made up of spindle-shaped cells [ 3 - 5 ].

The vascular spaces are large, lined by endothelial cells, and they may be filled with blood or thrombi [ 3 ]. The stroma is made up of spindle-shaped cells with short fascicles and numerous clear endothelial vacuoles resembling adipocytes [ 2 , 6 - 8 ]. Histopathologically, SCH mimics various other entities including, Kaposi’s sarcoma, pyogenic granuloma, epitheloid hemangioendothelioma, angiolipoma, and cavernous hemangioma (Table 2).

**Table 2:** Differential diagnoses of Spindle cell hemangioma [2,9- 13]

S.NO	Differential diagnosis	Differentiating features
1	Kaposi's Sarcoma	Kaposi's sarcomas generally show higher infiltrative pattern, high mitosis, cellular and nuclear atypia. Immunohistochemically, Kaposi sarcomas show positive expression for HHV-8. The spindle cells in Kaposi's sarcomas are usually positive for CD 34 marker, unlike SCH
2	Pyogenic granuloma	Pyogenic granulomas are reactive lesions that contain substantial amounts of inflammatory cells, while SCH is generally devoid of inflammatory infiltration. Spindle cell proliferation is not seen in pyogenic granuloma
3	Epitheloid hemangioendothelioma	Epitheloid hemangioendotheliomas have more solid architecture and less cavernous spaces unlike, SCH
4	Angiolipoma	Though the angiolipomas are vascular adipocytic tumors but they do not show spindle cell proliferations. However, the endothelial vacuoles in SCH may sometimes mimic adipocytes of angiolipomas
5	Cavernous hemangioma	Cavernous hemangiomas contain large vascular spaces and thrombi that resemble SCH, but they lack spindle cell proliferation

Various immunohistochemical studies have shown the positivity of the endothelial cells lining the vascular spaces for CD 31, CD 34, and ERG transcription factor , with the negative expression for the spindle cells. In the present case, also CD 31 and ERG transcription factor were found to be positive for endothelial cells and negative for spindle cells. 

Wang *et al*. [ 8 ] studied the expressions of D2 40, Prox 1 (expressed in endothelial cells of lymphatic channels), and WT 1 (Wilms Tumor) in 12 cases of SCH. They found the positive expression of the tumor cells for Prox 1, focally positive for D2 40 and negative expression of tumor cells for WT1. In the present study, also the expression of the tumor cells for WT1 was negative. The herpes virus 8 latent antigen 1 (HHV 8) is strongly associated with Kaposi's sarcoma [ 2 , 8 ]. 

In the present case, the HHV 8 expression for the tumor cells was negative excluding the possibility of Kaposi's sarcoma. The treatment of SCH is complete surgical removal and they do not tend to recur. In the present case, no recurrence was noted. The written consent of the patient was taken for the publication. 

## Conclusion

The diagnosis of SCH, solely based on histopathology is difficult, as it closely resembles Kaposi's sarcoma. The HHV 8 marker is useful to mark the difference between SCH and Kaposi's sarcoma.

## References

[1] Fletcher CD, Beham A, Schmid C ( 1991). Spindle cell haemangioendothelioma: A clinicopathological and immune histochemical study indicative of a nonneoplastic lesion. Histopathology.

[2] Savithri V, Suresh R, Janardhanan M, Aravind T ( 2022). Spindle cell haemangioma: Report of a rare entity. J Oral Maxillofac Pathol.

[3] Murakami K, Yamamoto K, Sugiura T, Kirita T ( 2018). Spindle cell hemangioma in the mucosa of the upper lip: a case report and review of the literature. Case Rep Dent.

[4] Weiss SW, Enzinger FM ( 1986). Spindle cell hemangioendothelioma: A low-grade angiosarcoma resembling a cavernous hemangioma and Kaposi's sarcoma. Am J Surg Pathol.

[5] Ide F, Obara K, Enatsu K, Mishima K, Saito I ( 2004). Rare vascular proliferations of the oral mucosa. Oral Surg Oral Med Oral Pathol Oral Radiol Endodontol.

[6] Lade H, Gupta N, Singh PP, Dev G ( 2005). Spindlecell hemangioendothelioma of the posterior pharyngeal wall. Ear Nose Throat J.

[7] Perkins P, Weiss SW ( 1996). Spindle cell hemangioendothelioma: An analysis of 78 cases with reassessment of its pathogenesis and biologic behavior. Am J Surg Pathol.

[8] Wang L, Gao T, Wang G ( 2014). Expression of Prox1, D240, and WT1 in spindle cell hemangioma. J Cutan Pathol.

[9] Chavva S, Priya MH, Garlapati K, Reddy GS, Gannepalli A ( 2015). Rare case of spindle cell haemangioma. J Clin Diagn Res.

[10] French KE, Felstead AM, Haacke N, Theaker J, Brennan PA, Colbert SD ( 2016). Spindle cell haemangioma of the tongue. J Cutan Pathol.

[11] Bajpai M, Pardhe N ( 2019). Report of a rare case of Epitheliod Hemangioendothelioma in tongue. J Dent (Shiraz).

[12] Yendluri DB, Chinta C, Vedula C ( 2023). Epithelioid Hemangioendothelioma of tongue: a rare presentation. J Dent (Shiraz).

[13] Bajpai M, Pardhe N, Kumar M ( 2018). Non-infiltrating angiolipoma of palate. Cukurova Med J.

